# An Unusual Presentation of Pauci-Immune Necrotizing Glomerulonephritis

**DOI:** 10.7759/cureus.12155

**Published:** 2020-12-18

**Authors:** Andrew Talon, Nina Tazi

**Affiliations:** 1 Internal Medicine, Creighton University School of Medicine, St. Joseph's Hospital and Medical Center, Phoenix, USA; 2 Internal Medicine, Dignity Health Medical Group, St. Joseph's Hospital and Medical Center, Phoenix, USA

**Keywords:** acute renal failure, encephalopathy, rapidly progressive glomerulonephritis, pauci-immune, vasculitis, mpo-anca, p-anca, renal-limited, crescentic glomerulonephritis, uremic encephalopathy

## Abstract

Renal-limited pauci-immune necrotizing glomerulonephritis presenting as acute encephalopathy is a rare occurrence. A 67-year-old Hispanic male presented to the hospital after being found down. He was found to have acute renal failure and high anion gap metabolic acidosis. Hemodialysis did not improve his encephalopathy. A vasculitis workup resulted in a high antimyeloperoxidase (MPO) antibody level. Renal biopsy revealed globally sclerotic glomeruli with focal thickened capillary loops, suggestive of pauci-immune necrotizing and crescentic glomerulonephritis (GN). Treatment consisted of high dose methylprednisolone and rituximab for induction, and three cycles of plasmapheresis, in addition to hemodialysis for uremia. Upon discharge, he was continued on hemodialysis and continued treatment with prednisone. Patients who present acutely with persistent uremic encephalopathy despite hemodialysis may warrant pursuing an alternative diagnosis, such as glomerulonephritis. Prompt diagnosis and treatment are necessary to improve the prognosis since untreated pauci-immune glomerulonephritis carries a high mortality rate.

## Introduction

Rapidly progressive glomerulonephritis (RPGN) is an uncommon cause of acute renal failure. Pauci-immune crescentic glomerulonephritis, the most common etiology of RPGN, typically presents with nonspecific manifestations of systemic inflammatory disease. Rarely it presents acutely with encephalopathy and acidemia. Untreated pauci-immune glomerulonephritis carries a one-year mortality of 80% [1]. Thus, timely biopsy and initiation of induction therapy are crucial to improve patient outcomes. Insights from this case may assist other clinicians in diagnosing atypical presentations of glomerulonephritis. 

## Case presentation

A 67-year-old Hispanic man presented to the emergency department after being found down by his roommate. He initially presented with lethargic consciousness and restlessness. He had no significant past medical history. He was previously prescribed risperidone and citalopram for unknown reasons. On examination, he did not have pitting pedal edema or facial puffiness. His blood pressure was 167/110 mmHg. Laboratory examination showed a total leukocyte count of 19.6 x 10^9^/L and hemoglobin of 8.6 mg/dL. The arterial blood gas showed pH 7.3, partial pressure of carbon dioxide (PaCO_2_) 21 mmHg, partial pressure of oxygen (PaO_2_) 118 mmHg, bicarbonate 10 mmol/L. Biochemical parameters were significant for high anion gap metabolic acidosis with an anion gap 28 mmol/L, serum creatinine of 17.4 mg/dL with unknown baseline, blood urea nitrogen (BUN) of 211 mg/dL, and serum albumin 2.9 mg/dL. Serum potassium was 4.4 mmol/L. Fractional excretion of sodium was 11.5% suggesting intrinsic or postrenal causes. Urinalysis showed microscopic hematuria, without hyaline, granular, white blood cell, or red blood cell casts on microscopy. Urine protein-to-creatinine ratio was 1.1 g/dL. Urine toxicology screen, methanol, and ethylene glycol were negative. Antinuclear antibody (ANA) was positive. C3 complement and C4 complement levels were 68 mg/dL and 14.9 mg/dL, respectively. Hepatitis and HIV serologies were negative. Anti-glomerular basement membrane (anti-GBM) and anti-double stranded DNA (anti-dsDNA) antibodies were negative. Antineutrophil cytoplasmic antibodies (ANCA) serology yielded an anti-myeloperoxidase (MPO) antibody level of 6.3 (normal level <1.0). Serum and urine protein electrophoresis (SPEP and UPEP) was noncontributory. Renal ultrasound showed no evidence of chronic renal disease, atrophy, or hydronephrosis.

**Figure 1:**
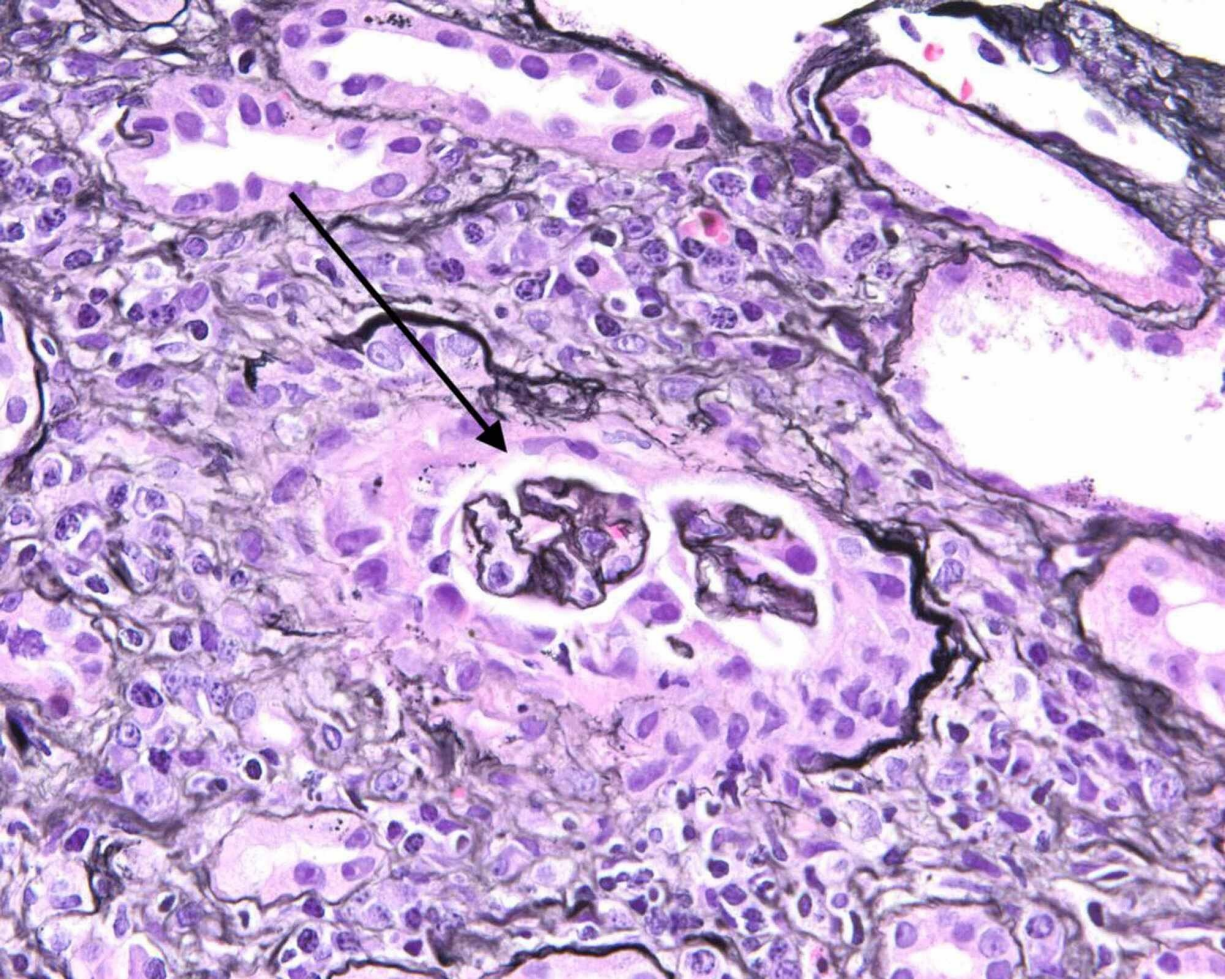
Light microscopy of renal biopsy: two of six non-sclerotic glomeruli showing cellular/fibrocellular crescent formation

**Figure 2:**
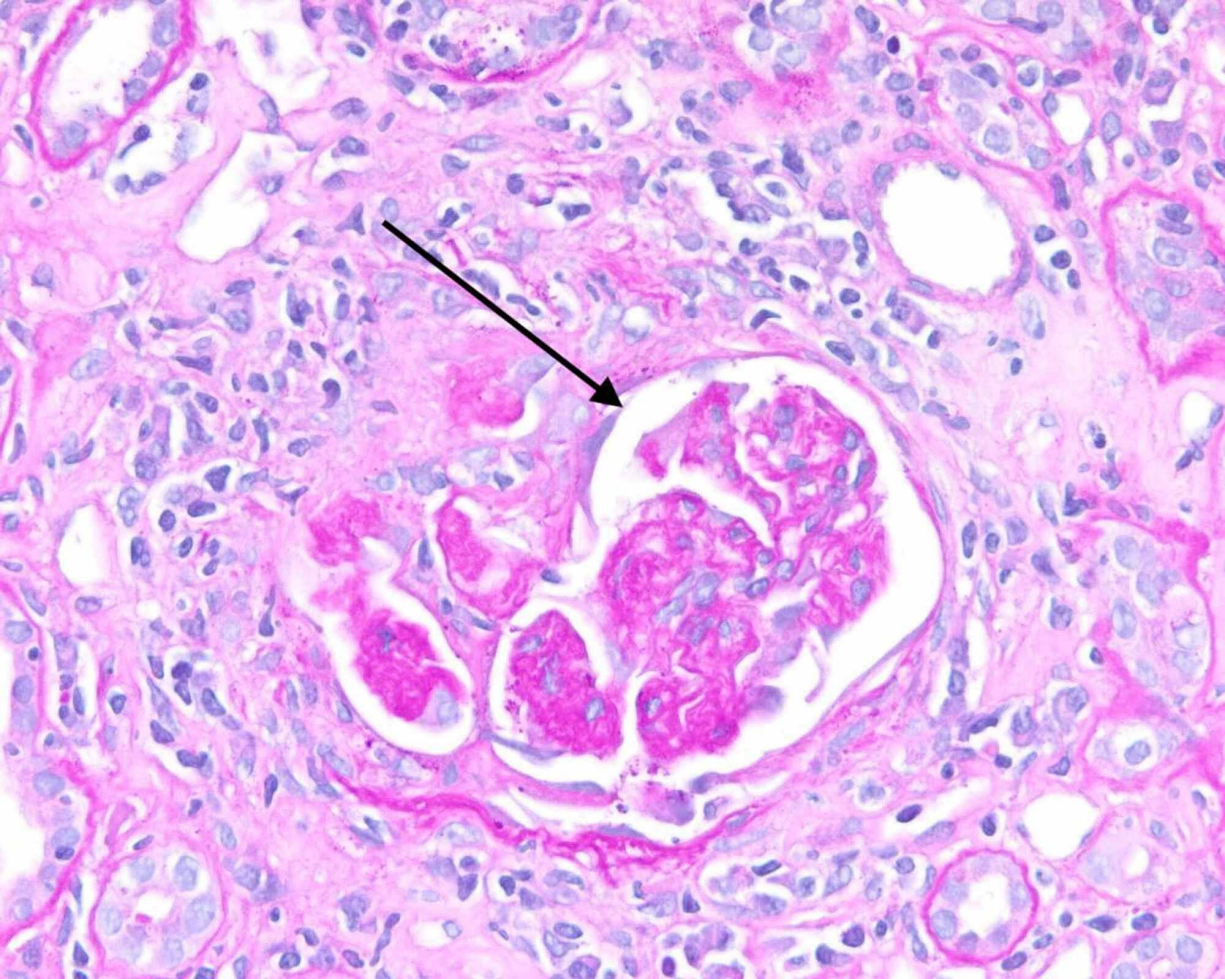
Light microscopy of renal biopsy: glomeruli with focal thickened capillary loops and ischemic wrinkling of the basement membrane (few holes) and periglomerular fibrosis; few with segmental sclerosis

**Figure 3:**
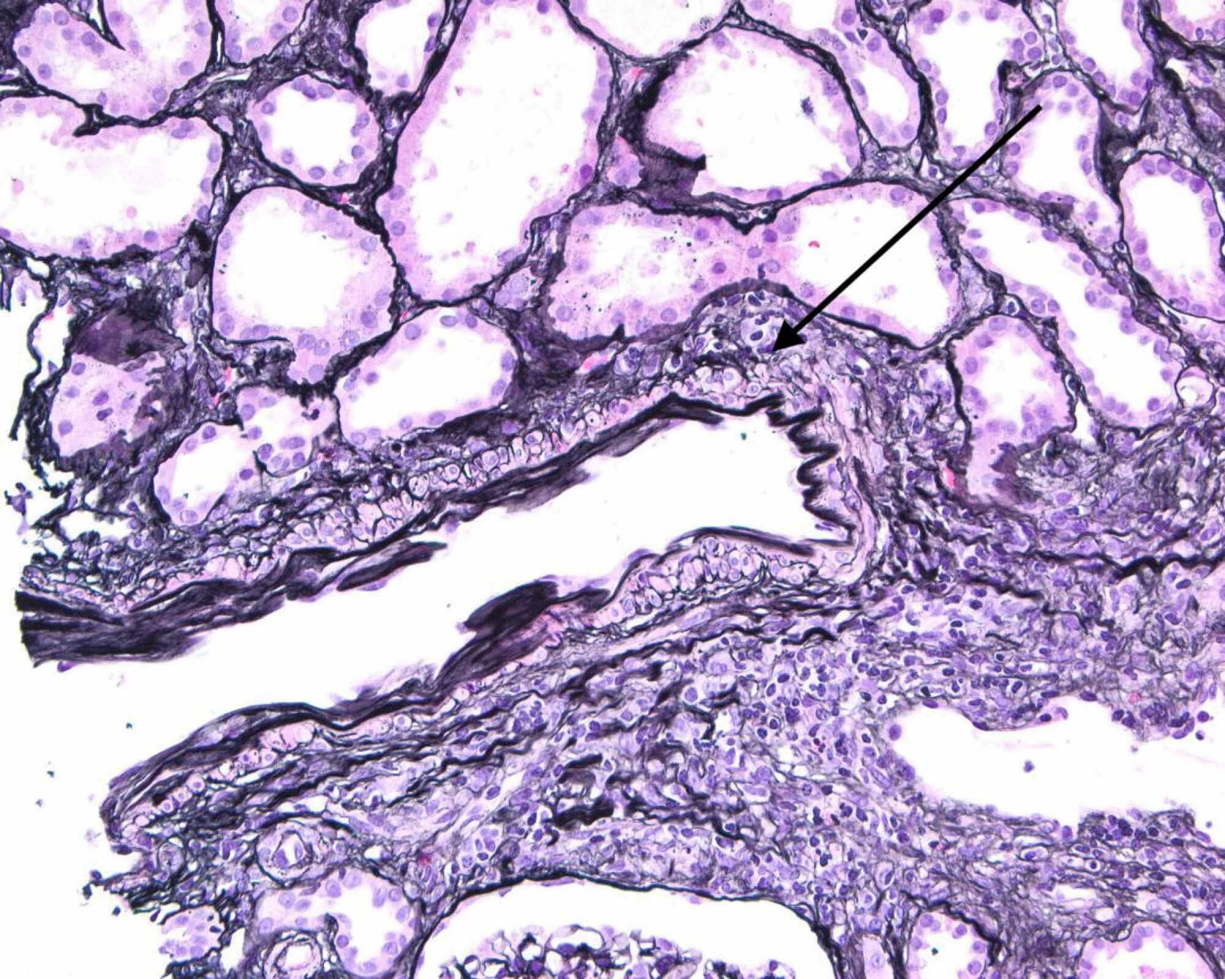
Light microscopy of renal biopsy: lymphocytic tubulitis, seen in crescentic glomerulonephritis. Intact tubules with reactive nuclei, thinning and isometric vacuolization

He was initiated on hemodialysis for severe acidemia and uremia. Mannitol was given during his initial dialysis session to reduce the risk of dialysis disequilibrium syndrome. Due to the lack of improvement following three dialysis sessions (persistently unable to follow simple commands, oliguria, despite improvement in serum creatinine from 17.4 mg/dL to 7.15 mg/dL), a renal biopsy was carried out to ascertain the nature of glomerular pathology. Renal biopsy showed crescentic glomerulonephritis (GN) pauci-immune type. Light microscopy showed globally sclerotic glomeruli with focal thickened capillary loops (Figure 1), severe interstitial fibrosis and tubular atrophy (Figure 2), and lymphocytic tubulitis (Figure 3). No immune type electron-dense deposits were seen in the basement membrane. The patient received induction therapy with 500 mg boluses of methylprednisolone, followed by prednisone 50 mg/day along with three sessions of plasmapheresis and the first dose of rituximab. Magnetic resonance imaging (MRI) of the brain showed no evidence of vasculitis. Encephalopathy markedly improved with induction therapy and was alert and oriented. Despite this, he did not recover his renal function, and he was discharged home on maintenance dialysis three times per week. Unfortunately, upon his anticipated discharge day, insurance was unable to approve his second dose of rituximab. He is currently considered to have the end-stage renal disease (ESRD). 

## Discussion

Eighty percent of patients with pauci-immune necrotizing glomerulonephritis (GN) present with renal-limited, myeloperoxidase-antineutrophil cytoplasmic antibody (MPO-ANCA) positive vasculitis [1]. Pauci-immune necrotizing GN is part of the spectrum of granulomatosis with polyangiitis (GPA) as they are both similar histologically [2]. Laboratory data from admission was more concerning for acute renal failure with severe metabolic acidosis, which is more commonly seen following toxic alcohol ingestions or acute tubular necrosis. The fractional excretion of sodium of 11.5% suggests that intrinsic or postrenal causes were more likely than prerenal causes. The ultrasound of the kidney and bladder did not reveal any obstruction, and the lack of monoclonal gammopathy on SPEP and UPEP made multiple myeloma unlikely. The presenting complaints in RPGN with crescentic GN may be a severe nephritic syndrome: hematuria, low urine output, hypertension, and edema [3]. More commonly, however, RPGN has an insidious onset, with the initial symptoms being fatigue or edema [2]. Our case is unique in that our patient presented encephalopathic. To our knowledge, this is the first case of pauci-immune necrotizing crescentic glomerulonephritis presenting with acute encephalopathy due to uremia. This serves as a diagnostic dilemma since the majority of our patients presenting encephalopathic with acute renal failure are more often due to acute tubular necrosis secondary to hemodynamic instability or toxic ingestion. The patient's unchanged mentation and minimal improvements in renal function parameters despite dialysis prompted us to pursue an extensive glomerulonephritis panel. Ultimately, a kidney biopsy is required even prior to the initiation of empiric treatment, even in highly suspicious patients since the regimen involves immunosuppression.

Primary and idiopathic RPGN is classified into five types based on immunoglobulin (Ig) deposition. Type I RPGN includes anti-glomerular basement membrane disease (linear IgG deposition) [3]. The most common etiologies of Type II RPGN (granular Ig deposition) include lupus nephritis, cryoglobulinemic membranoproliferative glomerulonephritis, and secondary membranous nephropathy. Type III RPGN, presenting with few or no immune deposits (pauci-immune), was the most relevant for this patient, which includes small vessel vasculitis such as microscopic polyangiitis (MPA), GPA, and eosinophilic granulomatosis with polyangiitis (EGPA). Type III RPGNs are more commonly associated in patients with high levels of antimyeloperoxidase antibodies (anti-MPO) or antiproteinase-3 antibodies (anti-PR3) [4]. Anti-MPO antibody is most commonly associated with MPA and EGPA [1].

Therapeutic guidelines in RPGN are not well established; however, the current standard is to treat with intravenous methylprednisolone for three days, followed by an oral prednisone taper, in combination with Cytoxan® (cyclophosphamide) or rituximab and plasmapheresis [5]. Our approach was to begin with steroids and plasmapheresis and to withhold rituximab until we received preliminary biopsy results. Following the completion of plasmapheresis, the patient was started on rituximab for immunosuppression. Rituximab was chosen over cyclophosphamide, given the risks of hemorrhagic cystitis associated with the latter. Despite this, rituximab is not FDA-approved for the treatment of RPGN, and the patient was unable to receive his second dose. 

## Conclusions

The diagnosis of RPGN is exceedingly rare. Untreated RPGN typically progresses to end-stage kidney disease over a period of days to weeks. Thus, prompt diagnosis and treatment are warranted to improve the prognosis. We recommend a full renal workup, including serologies, and if still inconclusive, renal biopsy. An accurate and urgent diagnosis is essential in the patient presenting with clinical findings suggestive of RPGN. 
